# 

**Published:** 1873-10

**Authors:** 


### Books & Periodicals.

GERMAN TEXT BOOKS.—We have received and have carefully examined, a series of books intended for those seeking a knowledge of the German language. They are published by E. Steiger, 22 & 24 Frankfort St., New York, and are entitled Ahn's First German Book, which teaches the letters of the alphabet, including script, and easy sentences in German in order to prepare the student for Ahn's Second German Book. This book teaches how to translate and speak the language, and the exercises are composed of sentences that are used in every day life, the language being natural, easy, and attractive.

Accompany these two excellent books is a key, which is of great assistance to the teacher as well as the private learner. Also a series of German Reading Charts giving German script letters, of a size, so as to be seen by a large class, this doing away with the necessity of writing them upon the black-boards, when instructing the class. Altogether these little books are by far the best we have yet examined, and we cheerfully recommend them to teachers and students.

THE SCIENTIFIC AMERICAN.—It seems superfluous to recommend this well known journal, as its undoubted excellence and usefulness is acknowledged by every one. It would seem indispensable to the mechanic and is an instructive and entertaining journal for the family. If you do not already take it, send your subscription to Munn & Co., Publishers, New York. Price $3.00 per year. Clubs with BISTOURY at same price.

THE ALDINE.—The only artistically illustrated journal published. The engravings contained in any single number are well worth a year's subscription. $5.00 per. year. Send your order to Jas. Sutton & Co., 58 Maiden Lane, New York.

PETERSON'S LADIES MAGAZINE, remains without a rival. All the ladies praise it as the best and cheapest magazine, of all that are published, for their use. Only $2.00 per. year. Chas. J. Peterson 300 Chesnut St., Phila.

BISTOURY & Peterson's Magazine, one year at same price.

THE ELMIRA

### A USEFUL PREMIUM.

For one hundred and ten subscribers to THE BISTOURY, at *Fifty Cents each*, amounting to $55,00, we will give a

**GROVER & BAKER SEWING MACHINE, COSTING $55 00**

Everybody knows what a GROVER & BAKER Machine is, so that it requires no puffing. We will guarantee them to be new, and in perfect and complete order.

For twenty subscribers, at fifty cents each, we will send to the getter up of the club, one of

**McAllister's New Microscopes,**

The price of which is Five Dollars.

For seventy subscribers we will give as a premium a good, silver, hunting case, AMERICAN WATCH, worth $35,00.

Those who prefer to work for money, can do so according to our

### CASH LIST.

Examine it and learn *How to make from ten to twenty dollars a day!*

| | | | |
|---|---|---|---|
| For 100 Subscribers | and $50,00 | you may retain | $25,00 |
| " 50 " | 25,00 | " " | 10,00 |
| " 25 " | 12,50 | " " | 5,00 |
| " 10 " | 5,00 | " " | 2,00 |
| " 5 " | 2,50 | " " | 1,00 |

So that you get your premiums in SOLID CASH, instead of cheap books, worthless pictures, or "pinched beck" jewelry.

**THE WAY TO REMIT TO US.**

Collect $50 from 100 Subscribers. Then put $25 of it in your own pocket for your work and buy a Post Office order or draft with the other $25, and remit it to us. Also, send the names and address, (Post Office, County and State,) of each subscriber, PLAINLY WRITTEN, when they will receive THE BISTOURY by return mail, which will be an evidence to them that your contract has been faithfully performed. For fifty subscribers you will send us $15, you retaining $10, and so on, down through the list.

Women, who are worn out with sewing, throw down your needles, take a copy of THE BISTOURY and solicit subscriptions from your neighbors. You will find it a healthier and more profitable employment.

Post Masters can, without trouble, secure large lists at their offices, and make it pay better than their salaries.

Young men, out of employment, can earn a suit of clothes in one day, and make their canvassing pay them ever afterwards.

Boys and girls can canvass for THE BISTOURY,

**AND MAKE MORE MONEY**

than they ever dreamed of possessing before.

Try it, everybody. There is money in it, and

**You have your Pay in your own hands.**

Send all sums over $5 by Post Office Order or Draft, made payable to THAD S. UP DE GRAFF, M. D. Smaller sums may be sent in currency.

☞ Those who desire to get up Clubs, will have sample copies sent them FREE, by writing for them. ADDRESS,

**THE BISTOURY,**
**ELMIRA, N. Y.**

### SUBSCRIPTIONS RECEIVED.

In this column we will credit every subscription received during the last quarter. Subscribers will please notify us immediately if their payments are not duly credited. The State only is given—this, with the full name of the party subscribing, will be sufficient to indicate who is meant:

NEW YORK.—M. E. Mead, W. M. Farrar, Mrs. P Caldwell, Dr. H. Hardy, Dr. A. Rice, Dr. F. M. Pasco, W. Fallett, S. W. Badger, Dr. J. O. Hill, Dr. E. Bigelow, J. M. Young, E. Osborne, Dr. L. Parker, Dr. Ed. Young, Dr. P. R. Spencer, Dr. Shearer.

PENNSYLVANIA.—Dr. J. H. Shearer, Dr. A. K. Minich, Henry Gibbs, Mrs. J. Forrest, Alex. Johnston, E. H. Stratton, H. C. McCarty, Wm. Stewart, Dr. John Pasco, Dr. L. Weiley. Dr. J. H. Shindel, Dr. R. Bergstresser.

INDIAN TERRITORY.—Dr. O. G. Given.

KANSAS.—Dr. Geo. T. Brown, Chas. H. Taylor, Mrs. S. A. Bush.

MICHIGAN.—Dr. J. W. Babbitt.

MASSACHUSETTS.—Dr. E. P. Hurd.

MISSISSIPPI.—Dr. S. G. Wellborro.

MISSOURI.—Capt. J. W. Allen.

ONTARIO, CANADA.—Dr. O. Yates.

**Carelessly Written Addresses**:—G. C. Box 97, Madison, N. J., or N. Y.,—which?

Dr. Everett or Everell, Harrisburg—no State; John Zimmer—no address.

Address, **THE BISTOURY,**
ELMIRA, N. Y.

